# Acetyl-cholinesterase-inhibitors slow cognitive decline and decrease overall mortality in older patients with dementia

**DOI:** 10.1038/s41598-022-16476-w

**Published:** 2022-07-16

**Authors:** Marco Zuin, Antonio Cherubini, Stefano Volpato, Luigi Ferrucci, Giovanni Zuliani

**Affiliations:** 1 Department of Translational Medicine, University of Ferrara, 44124 Ferrara, Italy; 2 Geriatria, Accettazione Geriatrica e Centro Di Ricerca Per L’invecchiamento, IRCCS INRCA, Ancona, Italy; 3 Department of Medical Sciences, University of Ferrara, Ferrara, Italy; 4 Translational Gerontology Branch, National Institute On Aging, National Institutes of Health, Baltimore, MD USA

**Keywords:** Cognitive ageing, Neurological disorders, Epidemiology, Outcomes research

## Abstract

We evaluated the effect of Acetyl-cholinesterase-inhibitors (AChEIs) on cognitive decline and overall survival in a large sample of older patients with late onset Alzheimer’s disease (LOAD), vascular dementia (VD) or Lewy body disease (LBD) from a real world setting. Patients with dementia enrolled between 2005 and 2020 by the "Alzheimer's Disease Research Centers" were analysed; the mean follow-up period was 7.9 years. A 1:1 propensity score matching was performed generating a cohort of 1.572 patients (786 treated [AChEIs +] and 786 not treated [AChEIs-] with AChEIs. The MMSE score was almost stable during the first 6 years of follow up in AChEIs + and then declined, while in AChEIs− it progressively declined so that at the end of follow-up (13.6 years) the average decrease in MMSE was 10.8 points in AChEIs- compared with 5.4 points in AChEIs + (*p* < 0.001). This trend was driven by LOAD (Δ-MMSE:−10.8 vs. −5.7 points; *p* < 0.001), although a similar effect was observed in VD (Δ-MMSE:−11.6 vs. −8.8; *p* < 0.001). No effect on cognitive status was found in LBD. At multivariate Cox regression analysis (adjusted for age, gender, dependency level and depression) a strong association between AChEIs therapy and lower all-cause mortality was observed (H.R.:0.59; 95%CI: 0.53–0.66); this was confirmed also in analyses separately conducted in LOAD, VD and LBD. Among older people with dementia, treatment with AChEIs was associated with a slower cognitive decline and with reduced mortality, after a mean follow-up of almost eight years. Our data support the effectiveness of AChEIs in older patients affected by these types of dementia.

## Introduction

Dementia is a major health problem in older populations, involving approximately 47 million worldwide^1^. In the U.S. the prevalence of dementia is about 15% in people over 68 years of age^2^, and Alzheimer’s disease (AD) represents the most frequent type, affecting 5.5 million people^2^; late-onset Alzheimer’s disease (LOAD) is the most common form of AD, with an onset over 65 years of age. Despite the recent and much-debated approval of Aducanumab by FDA for AD treatment, Acetyl-cholinesterase-inhibitors (AChEIs), such as donepezil, galantamine and rivastigmine, represent the first line pharmacological treatment options in patients with AD, with the aim of treating symptoms and slowing the natural course of the disease^3–5^. Although the efficacy of AChEIs has been evaluated in different randomized double-blind controlled trials (RCTs) conducted in subjects with AD^6–8^, as well as in patients affected by vascular dementia (VD)^9,10^, their efficacy in the real world setting has been questioned^7,11^. Doubtless, RCTs represents the “gold standard” method to evaluate the treatment outcomes^12^, but their results may be difficult to generalize in daily clinical practice due to the highly selected cohort included which only partially represents the real-life population^13,14^. Conversely, observational studies have the advantage of evaluating the effectiveness and safety of drugs in non-selected cohorts of patients. At present, only a few observational investigations have investigated how AChEIs treatment may influence cognitive decline in patients with LOAD or other forms of dementia^15–18^. However, definitive results were not obtained, especially concerning the effect of long-term treatment. Therefore, the aim of the present study was to evaluate the rate of cognitive decline, as well as the overall survival, in a large sample of patients affected by dementia, treated or not treated with AChEIs in a real-world setting.

## Results

### Characteristics of the study population

**Figure 1:**
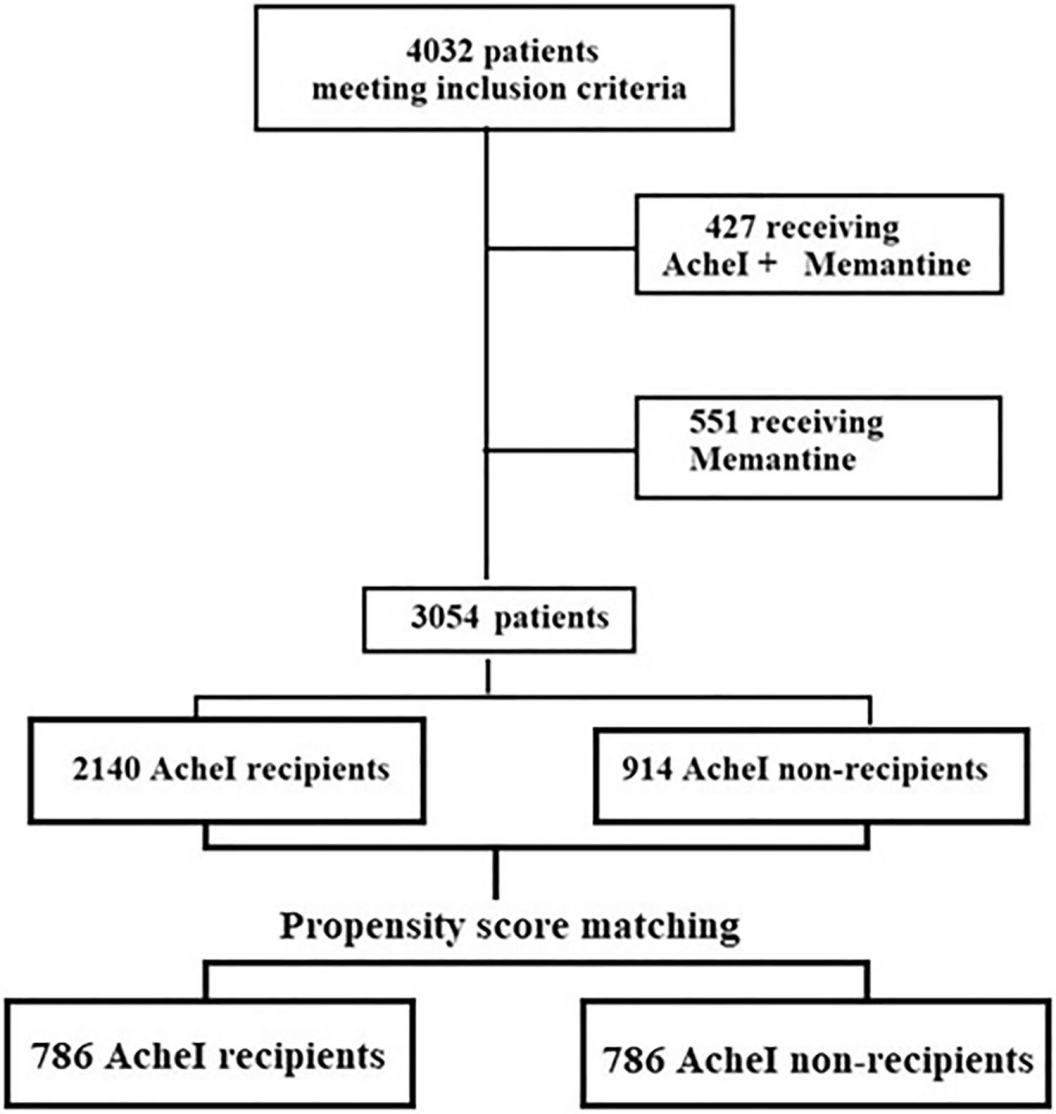
Flow chart of the study.

Overall, 3054 patients (1537 females, mean age 76.4 ± 5.6 years) met the inclusion criteria and were included into the study (Fig. 1). The baseline characteristics of the unmatched and matched groups and their relative medication use are presented in Table 1. In the entire cohort, LOAD resulted the first cause of dementia, followed by VD and LBD. As regards the AChEI + group, at baseline 62% of patients received donepezil, 24% rivastigmine and 14% galantamine. Before conducting the propensity score matching, AChEI− patients were older, more frequently males and required higher levels of assistance (all *p* < 0.001). Notably, the prevalence of diabetes and depression was higher in AChEI + patients (*p* < 0.001) (Table 1); moreover, these patients were more frequently treated with antidiabetic agents (*p* = 0.03), antidepressants (*p* < 0.001), and antipsychotics (*p* < 0.001), while received less frequently anxiolytic drugs (*p* = 0.01) compared with AChEI− patients. After the matching process, 786 patients were included into each group and, as expected, no more differences were observed. The mean follow-up period of the matched pairs was 7.9 ± 5.6 years [min: 2.3–max: 13.6 years].

**Table 1:** Principal characteristics and medications of patients with dementia treated or not with AcheIs, before and after matching.

	Before matching			After matching		
	AcheI + N = 2140	AcheI–N = 914	*p*	AcheI + N = 786	AcheI–N = 786	*p*
Age at baseline (years)	72.9 ± 5.7	76.5 ± 5.5	** < 0.001**	75.1 ± 4.3	75.1 ± 3.1	0.99
Males (%)	1011 (47.2)	506 (55.4)	** < 0.001**	400 (50.8)	405 (51.5)	0.78
Years of formal education (%)	15.08 ± 5.72	14.9 ± 6.05	0.57	14.4 ± 3.2	14.4 ± 2.8	0.99
**Dependence Level**						
Require assistance for complex activities (%)	618 (28.9)	470 (51.4)	** < 0.001**	339 (43.1)	336 (42.7)	0.87
Require assistance for basic activities (%)	131 (6.1)	104 (11.4)		62 (7.8)	58 (7.3)	0.70
**Dementia Type**						
LOAD (%)	1100 (51.4)	705 (77.1)	** < 0.001**	625 (79.5)	616 (78.3)	0.56
LBD (%)	127 (5.9)	79 (8.6)		60 (7.6)	64 (8.1)	0.69
VD (%)	913 (42.6)	130 (14.2)	** < 0.001**	101 (12.8)	106 (13.4)	0.68
**Comorbidities**						
AF (%)	179 (8.3)	68 (7.4)	0.40	49 (6.2)	53 (6.7)	0.55
Stroke (%)	148 (6.9)	50 (5.4)	0.12	43 (5.4)	45 (5.7)	0.79
TIA (%)	133 (6.2)	59 (9.6)	0.83	51 (6.4)	47 (5.9)	0.68
HTN (%)	1277 (59.6)	532 (58.2)	0.47	432 (54.9)	441 (56.1)	0.63
DM (%)	338 (15.7)	102 (11.1)	** < 0.001**	91 (11.5)	84 (10.6)	0.56
Hypercholesterolemia (%)	1204 (56.2)	517 (56.5)	0.87	454 (57.7)	443 (56.3)	0.57
Thyroid disease (%)	397 (18.5)	145 (15.8)	0.07	100 (12.7)	109 (13.8)	0.52
Depression (in the last two years) (%)	682 (31.9)	332 (36.3)	**0.01**	270 (34.3)	259 (32.9)	0.55
Urinary Incontinence (%)	345 (16.1)	159 (17.3)	0.41	116 (14.7)	112 (14.2)	0.77
Anti-Adrenergic agents (%)	212 (9.9)	89 (9.7)	0.86	66 (8.3)	78 (9.9)	0.27
Anxiolytic agents (%)	239 (11.2)	74 (8.1)	**0.01**	56 (7.1)	61 (7.7)	0.65
Anti-hypertensive drugs (%)	1287 (60.1)	533 (58.3)	0.37	481 (61.1)	496 (63.1)	0.41
Antidepressants (%)	513 (24.0)	328 (35.9)	** < 0.001**	263 (33.4)	254 (32.3)	0.64
Antipsychotic agents (%)	54 (2.5)	84 (9.1)	** < 0.001**	42 (5.3)	49 (6.2)	0.44
Antidiabetic agents (%)	260 (12.1)	87 (9.5)	**0.03**	68 (8.6)	74 (9.4)	0.58
Lipid-lowering medications (%)	991 (46.3)	756 (49.2)	0.14	390 (49.6)	401 (51.0)	0.57

*AF* Atrial Fibrillation, *HTN* Arterial Hypertension, *TIA* Transient ischaemic attack, *DM* Diabetes mellitus. *LBD* Lewy body dementia, *LOAD* Late onset Alzheimer disease. *VD* vascular dementia.

Significant values are in bold.

### ChEIs treatment and cognitive decline

**Figure 2:**
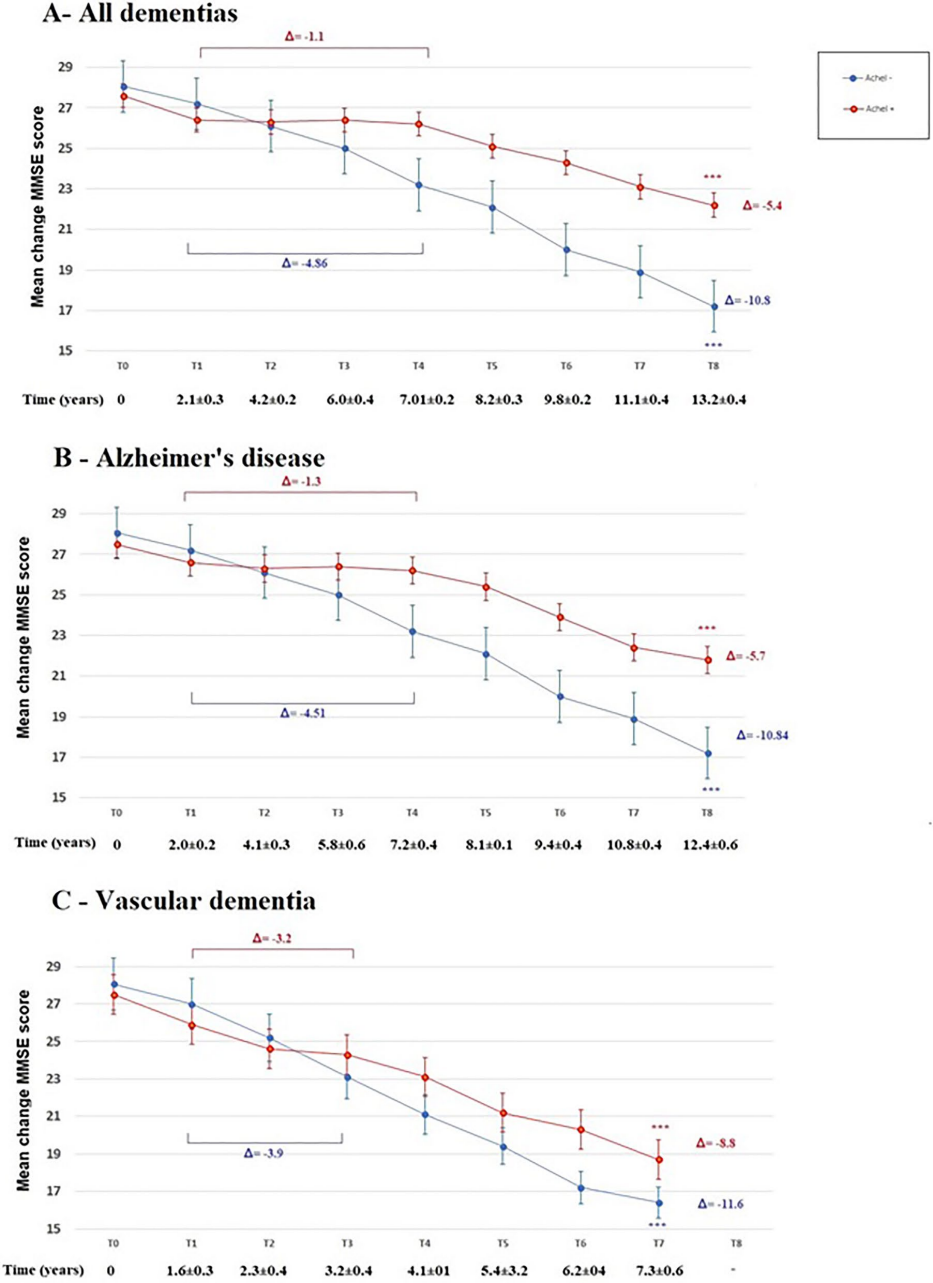
Mini Mental State Examination (MMSE) score during follow-up in all patients with dementia (**A**), LOAD (**B**), and vascular dementia (**C**) according to treatment with AChEIs. ***p < 0.001 for trend (Adjusted curves–for propensity score matched cohorts).

In the whole sample, the average MMSE score decreased in both groups over time (*p* for trend < 0.001). Specifically, the curve describing the descent of MMSE score in AChEI + dementia patients was almost stable during the first 6 years, while that of AChEI− patients progressively declined, so that the two curves began to diverge after 4 years of follow-up. In the following years, a greater reduction in MMSE score was observed in AChEI- patients; therefore, at the end of follow-up the average decrease in MMSE score in this group (Δ:−10.8 points) was much greater compared to that observed AChEI- patients (Δ:−5.4 points) (*p* < 0.001) (Fig. 2, Panel A).

This trend was substantially driven by the LOAD patients (Fig. 2, Panel B). Indeed, in this group the average MMSE score during the follow-up decreased in both groups (*p* for trend < 0.001), with a greater reduction at the end of follow-up in AChEI- patients (Δ:−10.8) compared to AChEI + (Δ:−5.4) (*p* < 0.001). The MMSE score in AChEI + was almost stable during the first 6 years, and the two curves began to diverge after 4 years of followup.

As reported in Supplementary Table 1, during the first 6 years of follow-up, most of LOAD AChEIs + patients displayed a stable/improved MMSE score; after that, MMSE progressively declined in all individuals.

The curves describing the descent of MMSE in patients with VD showed a different trend (Fig. 2, Panel C). The average MMSE score started to decrease immediately after baseline observation in both AChEI + and AChEI− patients; however, the two curves began to diverge after about 3 years, so that a greater cognitive decline was observed in AChEI− (Δ:−11.6) compared to AChEI + (Δ: −8.8) at the end of follow up (*p* < 0.001). In the VD group, most of patients treated with AChEIs displayed a stable/improved MMSE score only during the period of follow-up between 1.6 and 3.2 years; after that, MMSE substantially declined in all individuals.

As regards the few patients affected by LBD (n = 124 after matching), our analysis did not show any significant difference in the rate of cognitive decline based on presence or absence of the treatment with AChEIs (data not shown).

We performed additional analyses in order to examine the effect of AChEIs treatment on change over time in MMSE score. This was done by estimating the change in MMSE score over time according to AChEIs treatment (Supplementary Table 2). Overall, both groups of patients experienced a significant decline in MMSE score over the 8-year period (all *p* values < 0.001). However, the differences between the two groups became more pronounced as time progressed. In the fully adjusted random effect model, patients using AChEIs had an estimated average decline per year lower than those of patients not using AChEIs and the difference between the slopes (0.60 point per year) was statistically significant (*p* 0.002).

### AChEIs treatment and overall survival

**Figure 3:**
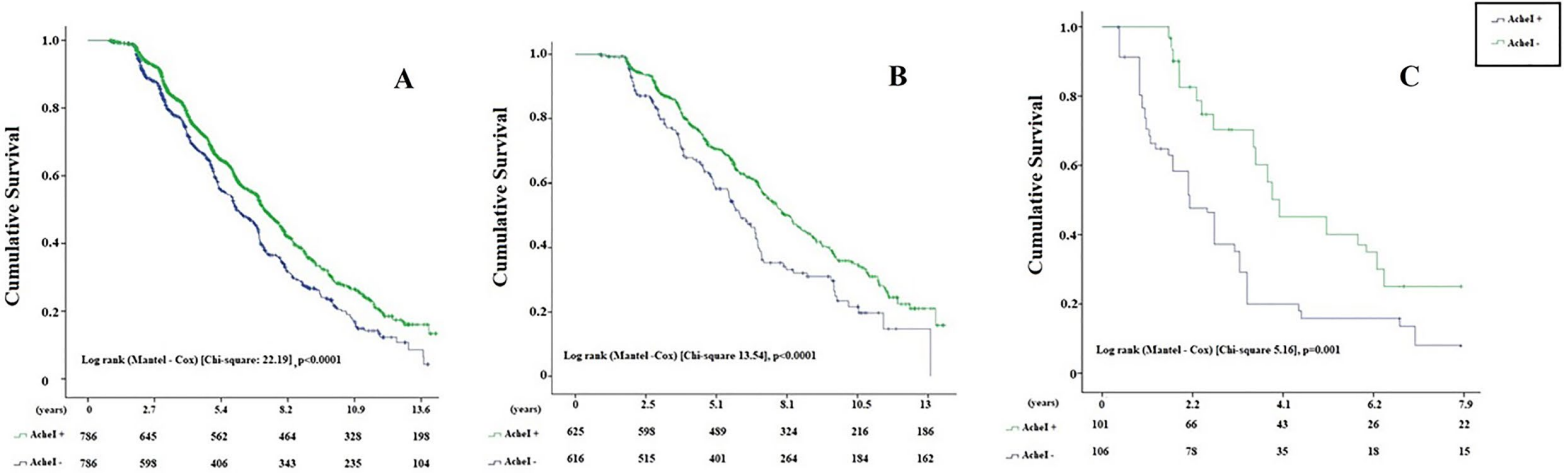
Cumulative survival (Cox multivariate regression analysis) after propensity score matching in patients with dementia treated or not treated with AChEIs: (**A**) all patients; (**B**) LOAD; (**C**): vascular dementia.

Kaplan–Meier curves comparing the overall survival in the whole sample (adjusted—after matching) between AChEI + and AChEI- patients (Fig. 3, Panel A), LOAD (Fig. 3, Panel B) and VD (Fig. 3, Panel C) demonstrated lower mortality rates in patients receiving the treatment (log-rank test, *p* < 0.0001 for all dementia and LOAD; *p* = 0.001 for subjects with VD).

At univariate Cox regression analysis, the treatment with AChEIs (HR: 0.61, 95%CI: 0.54–0.68; p < 0.0001), age (for 1 year increase, HR:1.04, 95%CI: 1.03–1.05, *p* < 0.0001), sex (males vs females, HR:0.64, 95%CI: 0.57–0.71, *p* < 0.0001), dependency level (HR:1.25, 95%CI: 1.20–1.30, *p* < 0.0001), and diagnosis of depression (HR: 1.32, 95% CI: 1.26–1.38, *p* < 0.0001) were independently associated with total mortality in all patients with dementia (unadjusted sample—before matching). At multivariate Cox regression analysis, after adjustment for age, gender, dependency level and depression and baseline MMSE, a strong association between AChEIs therapy and lower all-cause mortality was confirmed, also in sub-group analyses separately conducted in patients with LOAD and VD (Table 2). Similar significant results emerged in the few patients affected by LBD (Supplementary Table 3).

**Table 2:** Multivariate Cox regression analysis for overall mortality in all patients with dementia (Unadjusted—before Matching) (A) and in patients with LOAD (B) or VD (C).

	B	SE	Wald	HR	95% CI	*p*
**(A)**						
AChEI +	−0.522	0.56	86.89	0.59	0.53–0.66	< 0.0001
Age (years)	0.042	0.005	83.71	1.02	1.01–0.04	< 0.0001
Dependence Level	0.200	0.02	87.04	1.22	1.17–1.27	< 0.0001
Depression	0.277	0.024	129.04	1.31	1.25–1.38	< 0.0001
Gender (M vs. F)	−.465	0.054	75.14	0.62	0.56–0.69	< 0.0001
**(B)**						
AChEI +	−0.421	0.115	13.33	0.67	0.52–0.82	< 0.0001
Age (years)	0.047	0.006	60.55	1.04	1.03–0.06	< 0.0001
Dependence Level	0.128	0.029	19.54	1.13	1.07–1.20	< 0.0001
Depression	0.419	0.029	19.54	1.13	1.07–1.20	< 0.0001
Gender (M vs F)	−0.319	0.068	21.90	0.72	0.63–0.83	< 0.0001
**(C)**						
AChEI +	−0.277	0.59	22.04	0.75	0.67–0.85	< 0.0001
Age (years)	0.089	0.014	38.43	1.09	1.06–1.12	< 0.0001
Dependence Level	0.186	0.022	68.47	1.20	1.15–1.25	< 0.0001
Depression	0.419	0.141	66.47	1.52	1.15–2.00	0.003
Gender (M vs. F)	−0.386	0.059	43.39	0.67	0.60–0.76	< 0.0001

## Discussion

We evaluated the effect of the treatment with AChEI on the rate of cognitive decline and on long-term overall survival in a large sample of patients from the National Alzheimer’s Coordinating Center Uniform Data Set (NACC UDS). Indeed, the impact of these drugs in patients affected by dementia in the “real world” has not been systematically evaluated and has been sometimes questioned^7,11^. In this regard, the National Institute for Health and Care Excellence (NICE, UK) in its first technology appraisal guidance 111 published in 2006 (and successively modified in 2011 after strong pressures by relatives/caregivers’ organizations), gave a negative opinion on the use of AChEI in patients with dementia. The question of the real effectiveness of AChEI is of great interest especially in view of the recent and discussed approval by FDA of Aducanumab for the treatment of AD.

A first data emerging from our study is that AChEIs were preferentially prescribed to “younger” and female patients with dementia, demonstrating a better performance in the basic and complex activities of daily living. This may depend on the fact that these drugs are approved for the treatment of the mild-moderate stages of AD only, but might also highlight an ageist attitude or a selection bias. For this reason, we matched the patients treated and not treated with AChEIs in order to obtain two comparable groups.

### AChEIs treatment and cognitive decline

All patients with dementia treated with AChEIs showed a significant slower rate of cognitive decline (as measured by MMSE, although we noted some differences between LOAD, VD and LBD. In LOAD, AChEIs substantially stabilized the cognitive performance for a period of about 6 years after baseline, while in VD a significant slowing down of the cognitive function was observed only after about two years of treatment. It should be noted the faster rate of cognitive decline observed in VD compared with LOAD subjects, in good agreement with literature data^19,20^.

Currently, AChEIs are indicated only for the treatment of AD. On the contrary, their use in VD is considered “off label”^21,22^, although their efficacy has been suggested also in this type of dementia^9,10^ and it seems to be confirmed, also on long term period, by our study. Based on our results, patient with VD should be offered treatment with AChEIs also in the absence of comorbid AD, LBD or Parkinson’s disease dementia, as currently suggested by the National Health System (NHS, UK) and FDA (U.S.)^21,22^. On the other hand, AChEIs seem to be ineffective, as regards a possible effect on cognitive decline, in patients with LDB; however, we have to underline that the small size of the sample might have prevented the achievement of statistical significance. This result indirectly supports the conclusions of Rolinski et al. (Cochrane database review)^23^, who concluded that the effect of AChEIs in DLB remains unclear, rather than those of Matsunaga et al.^24^, who found a beneficial effect of AChEIs for LBD treatment.

In our LOAD cohort, the MMSE decline was slower compared to similar previous studies with AChEI^25^. However, we have to consider that: (a) the model of Mendiondo et al.^26^ reports a drop of 1.45 point per year in AD patients with MMSE score 24/30; since our patients started from an average MMSE over 26/30, we could expect an even smaller decline; (b) other studies from “real world” reported a slower MMSE decline compared to the results of Xu et al.^27–29^; (c) most important, previous studies mainly enrolled patients with moderate/severe AD, and this is associated with a faster MMSE decline^26^. Indeed, the baseline MMSE of our NAAC patients was much higher when compared with other cohorts, and we mainly included patients with mild form of dementia (79% had a MMSE ≥ 26/30.). This was probably due to: (a) early diagnosis of dementia among the NAAC clinics, due to different reasons (e.g. advanced diagnostic tools, increased awareness of population sensitized about cognitive decline); (b) exclusion of patients prematurely lost at follow-up (due to too short follow-up and partial lack of data) which means “accidental” exclusion of patients with more advanced form of dementia. Compared to those included in the final sample, patients excluded from the analysis had lower MMSE score and lower rate of AChEI treatment (51.5% vs. 70%), confirming that a higher number of patients with poor cognitive performance and not treated with ACheEI were excluded.

The evolution of cognitive decline and dependency before the treatment were not taken into account in our study; of consequence, other factors conducting the patient (or the physician) to accept AChEIs treatment were not considered (e.g. financial problem, last treatment failure, tolerance for side effects, personal believes, etc.). This  is an important point in order to enforce the design and correct use of clinical registries for future confirmation studies. Indeed, although only RCTs could really obtain comparable groups, it is almost impossible to organize such a long RCTs, especially in older patients with dementia.

### AChEIs treatment and overall survival

We also evaluated the effect of AChEIs on long-term survival in patients affected by dementia. Different parameters appeared to be independently associated with survival at multivariate analysis, including age, sex, levels of dependency and diagnosis of depression. These factors predicted survival not only in the whole sample but also in LOAD, VD and LDB groups, separately. With regards to the treatment with AChEIs, it was associated with a substantial reduction of total mortality in the entire cohort, with a 40% reduction observed in all patients, 33% in LOAD, 25% in VD, and 62% in LDB (all *p* < 0.001).

As shown by the Kaplan-Meyer curves, the effect of AChEIs on survival was observed after a period of about 2 years in the whole sample as well as in LOAD patients, while in VD patients AChEIs appeared to have an early effect on survival, soon after starting the treatment.

Our findings strengthen the results of other sporadic observational studies reporting lower mortality rates in patients with dementia treated with AChEIs. By analyzing data from the Swedish Dementia Registry (SveDem—mean follow-up: 17 months), Nordstrom et al. found a significant reduction of mortality (HR: 0.64) after multivariate analysis and matching^30^. Mueller et al. found a reduction of the risk of death by more than 20% in AD patients treated with AChEIs, after a median period of follow-up of 3 years^31^. More recently, Xu et al. compared a large sample of patients with Alzheimer’s dementia from the SveDem (mean follow-up: 5 years). These authors found that the use of AChEI was associated with a 27% lower risk of death (H.R.: 0.73) compared with non-users (30). However, previous studies evaluated a shorter follow-up, and no study had the opportunity of following the patients for a mean period of 7.9 years, as we did. Thus, our results are very similar in magnitude to those previously published. It is not known why AChEI treatment could increase survival in patients with dementia, and several mechanisms have been proposed including: (a) better compliance of patients actively treated with AChEI in life-style, diet, and management of their medical problems (even better caregiving); (b) AChEIs might reduce behavioral and psychological symptoms of dementia (BPSD), as suggested by some authors^32^, and this in turn would reduce the use of antipsychotic drugs and related mortality; (c) AChEIs may reduce peripheral cytokine production and increase vagal nerve activity^30^, and this has been associated with reduced cardiovascular mortality; (d) the slowdown of cognitive decline obtained by AChEIs therapy might contribute to the slowdown frailty progression thus reducing mortality rates. Finally, it might depend on “confounding by indication” bias, since healthier subjects are more often prescribed AChEI; however, after propensity score matching no differences emerged in the case–control cohorts.

### Limitations

We must acknowledge some important limitations of the study. 1. Participants comprising the NACC dataset represent a convenience sample, including clinical-referrals and community-based U.S.; of consequence, the NACC sample are not representative of all the U.S. population, partially reducing the generalizability of our results. 2. We excluded patients with short follow-up (< 2 visits; most had incomplete data); in this way we excluded many patients with moderate-severe dementia, and this might have influenced the rate of MMSE decline. However, since we have excluded more not-treated patients compared to treated-patients, this should have no effect on the association between treatment and MMSE change. 3. Despite performing the propensity score matching, the effects of some residual confounding may have results in not firm conclusions. 4. Considering the general clinical stability of patients after the diagnosis of dementia, we cannot exclude that these patients were evaluated much earlier compared to the standard of care applied in other Countries. 5. The comorbidities observed may be different from those affecting patients with dementia in other regions of the world, partially limiting the generalizability of our findings. 6. We cannot exclude that some of AD patients had a rapidly progressive Alzheimer’s disease, which may have influenced the cognitive decline during the follow-up period; indeed, previous investigations have estimated this phenotype in about 17% of subjects with mild AD^33,34^.

## Conclusions

Our results, obtained from the National Alzheimer’s Coordinating Center Uniform Data Set (NACC UDS), suggest that older people with dementia who are prescribed AChEIs have a slower decline in cognitive performance and a reduced mortality (by approximately 40%) after a follow-up of almost eight years. Basically, an early stabilization of cognitive performance, followed by a reduction in total mortality was observed in LOAD; in contrast, an early reduction in overall mortality and a subsequent slowdown in cognitive decline was observed in VD. Among subjects with LBD, the effect of AChEIs was negligible as regards cognition, but an independent effect of AChEIs on overall survival was observed. Our data support the effectiveness of AChEIs in older patients affected by these types of dementia.

## Methods

### Population

Data have been obtained from the National Alzheimer’s Coordinating Center Uniform Data Set (NACC UDS), which is a nationwide repository for longitudinal data collected from approximately 34 current or previously NIA funded Alzheimer's Disease Research Centers (naccdata.org). In the present investigation, data collected between 2005 and June 2020 were analysed. Patients were included into the present study if: (I) they were aged ≥ 65 years at baseline; (II) they had a diagnosis of dementia, including LOAD, vascular dementia (VD) or Lewy body disease (LBD); (III) they had the baseline visit and at least two in-person visits with evaluation of MMSE with two years of follow-up; (IV) the MMSE score was ≥ 10/30 (mild to moderate dementia). Conversely, participants were excluded if: (I) they had less than three in person-visits and/or relative MMSE consecutive evaluations; (II) they were receiving memantine; (IV) the MMSE was < 10/30 (severe dementia—AChEI are not indicated). The baseline registration in NACC registry was initiated at the time of the dementia diagnosis when the treatment started. Comorbidities and functional status were evaluated at baseline (time 0) by the medical team of the clinic by means of medical history, medical records, medical examination, and blood chemistry tests.

Local ethics committees at each of the sites approved the study, and all participants provided written informed consent. All procedures were performed in accordance with the relevant guidelines and regulations.

### Dementia’s definitions

Dementia was defined as meeting criteria for AD^35^ or VD or LBD^36,37^ defined as (1) objective cognitive impairment (i.e., performances falling greater than 1.5 standard deviations outside the age-adjusted normative mean) in at least 2 cognitive systems (memory, language, attention or executive functioning); and (2) cognitive impairment contributes directly to impaired activities of daily living.

Patients receiving available AChEIs (donepezil, rivastigmine or galantamine) were labelled as AChEIs recipients (ACheIs +). The ACheIs exposure was assumed to be constant.

### Cognitive decline evaluation

The Mini Mental State Examination (MMSE), which represents the most widely used cognitive assessment tool in dementia^38^ has been administered during the outpatient visits to evaluate the rate of cognitive decline over time in AChEI + and non-recipients (AChEI−). As known, MMSE uses a 30-point score to assess orientation, short-term and delayed recall, calculations, language interpretation, naming, and praxis. Higher scores represent better cognitive performance.

### Estimation of propensity scores

Before starting the analysis, 1965 patients were excluded (996 AD, 786 VD, 183 LBD) since they had a follow-up shorter than two years (most had incomplete data too) thus compromising the estimation of MMSE decline and mortality rates over time. These patients were older (mean age 82.1 ± 6.3 years), and 63.8% were males. MMSE score at baseline and after one year was available only in 40.4% (n. 787, mean MMSE 14.2/30) and 21.6% (n. 431, mean MMSE 11.3/30) of cases, respectively. Mortality data for these patients were available only for 6.5% of the cases. 51.5% of excluded patients assumed AChEI, compared to 70% of patients included into the study.

The population of interest included 3054 individuals with a mean MMSE score of 26.8/30; in more detail, 79% had a MMSE score ≥ 26, 16% had a MMSE between 20/30 and 26/30, and only 5% had a MMSE < 20/30. They were firstly divided into two groups, based on the presence or absence of a treatments with AChEI. Subsequently, due to differences in baseline covariates between AChEIs users and non-users, a 1:1 propensity score matching was performed. Specifically, each AChEI + patient was matched with an AChEI- patient with a similar propensity score, based on nearest-neighbour matching without replacement, using a caliper width equal to 0.1 of the SD of the logit of the propensity score, which indicate balance in covariates between groups^39^.

Based on this, a propensity score-matched cohort of 1.572 patients (786 AChEIs + and 786 AChEIs−) was generated, using a non-parsimonious multivariable logistic regression model to calculate, for each patient, a propensity score, using all covariates shown in Table 1, mean MMSE and mean follow-up length.

Compared with subjects included into the study after propensity score matching, the subjects excluded (n. 1482) were younger (72.8 ± 3.1 vs. 75.1 ± 2.8; *p* < 0.001), more frequently affected by VD (56.4% vs. 13.1%), less frequently affected by LOAD (38% vs. 78.9%), had a much lower average MMSE score (13.5 ± 3.1 vs. 27.6 ± 0.9; 45.1% of them ad a MMSE score < 10/30), and more frequently required assistance in basic activities of daily living (17.3% vs. 7.6%) (all *p* < 0.001).

### Statistical analysis

Pearson Chi-square and Wilcoxon rank-sum tests for the pre-matched population, and McNemar’s test and paired sample t-test for the matched population were used to compare baseline characteristics between the two groups as appropriate.

Based on the propensity score matching (PSM) method, the clinical baseline data of the two groups were balanced, and the regression model variables included age, sex, previous diabetes mellitus (DM), hypertension, stroke, smoking, alcohol consumption and systolic blood pressure (SBP), diastolic blood pressure (DBP) and heart rate of the patients after inclusion. The propensity score of each patient was calculated by the 1:1 nearest matching method, and caliper matching was employed to limit the logarithmic standard deviation of the propensity score to 0.10 to prevent the difference between each pair of matched individuals. Based on this, a propensity score-matched cohort of 1.572 patients (786 AChEIs + and 786 AChEIs−) was generated, using a non-parsimonious multivariable logistic regression model to calculate, for each patient, a propensity score, using as covariates: age at baseline (years), sex, years of formal education), dependence level, requirement of assistance for complex and basic activities, dementia type, comorbidities (atrial fibrillation, stroke/TIA, hypertension, diabetes, hypercholesterolemia, thyroid disease, depression in the last two years, urinary incontinence, anti-adrenergic agents, anxiolytic agents and anti-hypertensive drugs, antidepressants, antipsychotic agents, antidiabetic agents, lipid-lowering medications, baseline MMSE, and length of follow-up.

Difference in cognitive trajectories over time (MMSE score change) between patients using AChEIs and those not using AChEIs were estimated using mixed-effects repeated measures models of unstructured-variance–covariance matrix. To formally test the hypothesis of different MMSE slops over time the interaction term “time*AChEI treatment was fitted in the model. The model was adjusted for age, sex, education, number of medical follow-up visits and follow-up duration.

Multivariate Cox proportional hazards models were used to estimate the adjusted hazard ratios (HRs) and relative 95% confidence intervals (95%CI) for all-cause mortality in AChEIs + compared to AChEIs− in the unadjusted sample (before propensity score matching—whole sample, LOAD, VD, and LBD). Proportionality assumptions were examined through visual inspection of log–log survival curves, and analytical assessments using covariates-by-time interactions in the Cox model.

The association between AChEIs administration and all-cause mortality during the follow-up period was graphically evaluated using the Kaplan–Meier method. Subgroup analyses were also performed to examine the association between AChEIs administration and mortality among patients with LOAD and vascular dementia. Statistical significance was defined as *p* < 0.05. Statistical analyses were performed using SPSS package version 20.0 (SPSS, Chicago, IL, USA).

## Supplementary Information

Supplementary Table 1.

Supplementary Table 2.

Supplementary Table 3.
