# Supplementary material for: Acetyl-cholinesterase-inhibitors slow cognitive decline and decrease overall mortality in older patients with dementia
Source: Sci Rep. 2022 Jul 16;12:12214. doi: 10.1038/s41598-022-16476-w (PMC9288483; doi:10.1038/s41598-022-16476-w)
Supplement: Supplementary file 1 — Supplementary Table 1. [file 41598_2022_16476_MOESM1_ESM.docx]

**Supplementary Table 1:** modification of cognitive performance in patients treated with AChEIs in the first part of follow-up (T0-T4). LOAD: Late onset Alzheimer’s disease; VD: Vascular Dementia.

|  | **Cognitive performance**  **(MMSE)** | **T0-T1**  **(years 0-2)** | **T1-T2**  **(years 2-4.1)** | **T2-T3**  **(years 4.1-5.8)** | **T3-T4**  **(years 5.8-7.2)** |
| --- | --- | --- | --- | --- | --- |
| **LOAD** | **Improved or**  **Stable** | 51% | 64% | 82% | 60% |
|  | **Worsened** | 49% | 36% | 18% | 40% |
|  | | | | | |
|  | **Cognitive performance**  **(MMSE)** | **T0-T1**  **(years 0-1.6)** | **T1-T2**  **(years 1.6- 2.3)** | **T2-T3**  **(years 2.3-3.2)** | **T3-T4**  **(years 3.2-4.1)** |
| **VD** | **Improved or**  **Stable** | 43% | 54% | 58% | 49% |
|  | **Worsened** | 57% | 46% | 42% | 51% |
