# Supplementary material for: Acetyl-cholinesterase-inhibitors slow cognitive decline and decrease overall mortality in older patients with dementia
Source: Sci Rep. 2022 Jul 16;12:12214. doi: 10.1038/s41598-022-16476-w (PMC9288483; doi:10.1038/s41598-022-16476-w)
Supplement: Supplementary file 2 — Supplementary Table 2. [file 41598_2022_16476_MOESM2_ESM.docx]

**Supplementary Table 2.** Estimated difference in cognitive performance change (Mini-Mental-State-Examination scores (point/year) comparing ACheIs recipients and ACheIS not recipients.

|  | Unadjusted | | | Adjusted* | | |
| --- | --- | --- | --- | --- | --- | --- |
|  | ***MMSE***  ***Change per year*** | ***SE*** | ***p*** | ***MMSE***  ***Change per year*** | ***SE*** | ***p*** |
| All dementias | -0.60 | 0.23 | 0.002 | -0.56 | 0.24 | 0.01 |
| AD | -0.92 | 0.21 | 0.001 | -0.88 | 0.31 | 0.02 |
| VD | -0.38 | 0.19 | 0.04 | -0.34 | 0.18 | 0.04 |
| LBD | -0.48 | 0.32 | 0.61 | -0.43 | 0.29 | 0.58 |

MMSE: Mini-Mental-State-Examination; AD: Alzheimer’s disease; VD: Vascular dementia; LBD: Lewy Body dementia.

*Models adjusted for age, sex, education, follow-up duration and number of follow-up visits.
