# Supplementary material for: Acetyl-cholinesterase-inhibitors slow cognitive decline and decrease overall mortality in older patients with dementia
Source: Sci Rep. 2022 Jul 16;12:12214. doi: 10.1038/s41598-022-16476-w (PMC9288483; doi:10.1038/s41598-022-16476-w)
Supplement: Supplementary file 3 — Supplementary Table 3. [file 41598_2022_16476_MOESM3_ESM.docx]

**Supplementary Table 3:** Multivariate Cox regression analysis for all-cause mortality in patients affected by Lewy Body Dementia.

|  | B | SE | Wald | HR | 95% CI | p |
| --- | --- | --- | --- | --- | --- | --- |
| AChEI+ | -0.490 | 0.056 | 76.82 | 0.38 | 0.31-0.43 | **<0.0001** |
| Age (years) | 0.049 | 0.005 | 105.717 | 1.05 | 1.04-1.06 | **<0.0001** |
| Dependence Level | 0.224 | 0.021 | 118.11 | 1.25 | 1.20-1.30 | **<0.0001** |
| Depression | 0.052 | 0.029 | 3.28 | 1.05 | 0.99-1.11 | **0.07** |
| Gender (M vs F) | -0.441 | 0.056 | 61.69 | 0.64 | 0.57-0.61 | **<0.0001** |
